# Clinicopathological features and prognosis of breast cancer combined with symptomatic bone marrow metastases: A 10‐year, single‐center, real‐world study of 67 cases

**DOI:** 10.1002/cam4.5827

**Published:** 2023-03-23

**Authors:** Limin Niu, Huimin Lv, Mengwei Zhang, Huiai Zeng, Shuzhen Fu, Shude Cui, Zhenzhen Liu, Min Yan

**Affiliations:** ^1^ Department of Breast Disease, Henan Breast Cancer Center The Affiliated Cancer Hospital of Zhengzhou University & Henan Cancer Hospital Zhengzhou China; ^2^ Department of Clinical Laboratory The Affiliated Cancer Hospital of Zhengzhou University & Henan Cancer Hospital Zhengzhou China

**Keywords:** bone marrow metastases, breast cancer, clinicopathological, prognosis

## Abstract

**Purpose:**

Bone marrow metastasis (BMM) is uncommon in breast cancer (BC), and early diagnosis is challenging. BMM lacks definitive treatment options and poses a great threat to the survival of patients. Herein, we investigated the clinical features, prognosis, and factors affecting the prognosis of BC patients with symptomatic BMM to help improve the understanding of this disease and provide effective diagnostic and treatment strategies.

**Methods:**

Clinical data of 67 patients with BC and BMM were retrospectively analyzed for clinical characteristics, treatment, and prognosis of BMM. Univariate and multivariate analyses were performed to determine factors affecting overall survival following BMM (BMMOS).

**Results:**

Among patients with BMM, 86.6% were diagnosed after bone metastasis (BM), while 13.4% were diagnosed simultaneously with BM. A total of 73.1%, 13.4%, and 13.4% of the patients had hormone receptor‐positive/human epidermal growth factor 2‐negative (HR+/HER2−) tumors, HER2+ tumors, and triple‐negative tumors, respectively. The most common symptoms of BMM were the coexistence of anemia and thrombocytopenia (26.9%), anemia (19.4%), and pancytopenia (17.9%). The median BMMOS was 7.6 months (95% CI, 3.9–11.3). Univariate and multivariate analyses showed that BMMOS was associated with platelet count <75 × 10^9^/L at the time of BMM diagnosis. The BMMOS of patients who underwent endocrine therapy, combined chemotherapy, and mono‐chemotherapy after BMM was 15.7, 9.7, and 8.6 months, respectively, whereas that of untreated patients was 2.9 months, and the difference among the results was statistically significant (χ^2^ = 20.102, *p* < 0.0001). Changes in patient hemogram and/or body temperature during treatment were consistent with the overall effect of the disease (*p* < 0.0001).

**Conclusion:**

BMM should be considered in BC patients with BM, an unexplained reduction in hemogram parameters, especially anemia and thrombocytopenia, and/or fever without chills. Active, effective, individualized treatment strategies can prolong BMMOS.

## Graphical abstract

HR+/HER2− type tumors are more prone to bone marrow metastases (BMM), which occurs after or at the same time as bone metastasis (BM). BMM should be considered in breast cancer patients with BMs, an unexplained reduction in hemogram parameters (especially anemia and thrombocytopenia), and/or fever (without chills). Active, effective, individualized treatment strategies can prolong the BMMOS.

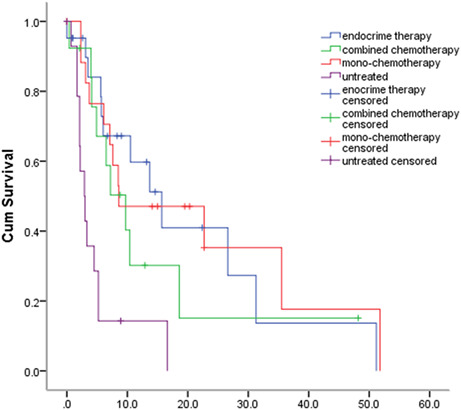

## 1 INTRODUCTION

In 2020, breast cancer (BC) replaced lung cancer as the most commonly diagnosed cancer among women worldwide.^1^ Bone metastasis (BM) accounts for approximately 65%–75% of metastatic BC (MBC) cases.^2^ In contrast to BM, the development of symptomatic bone marrow metastasis (BMM) is a rare event during MBC. The reported incidence of BMM is only 0.17% in MBC^3^ and 0.6%–1.7% in solid tumors.^4^, ^5^

BMM is uncommon in BC. It signifies the invasion of BC cells into the bone marrow, which may damage bone marrow hematopoietic stem cells and lead to repetitive fever, progressive anemia, and thrombocytopenia. The degree of bone marrow infiltration leading to manifestations of BMM is complex and diverse. The current diagnosis of BMM relies mainly on bone marrow aspiration smears and biopsies. In particular, bone marrow trephine biopsy was found to be the most sensitive technique for the detection of BMM.^4^ However, bone marrow aspiration smear and trephine biopsy are not routine clinical practices. Hence, the early diagnosis of BMM is limited by the lack of specific clinical manifestations^3^, ^4^, ^5^, ^6^, ^7^, ^8^, ^9^, ^10^ and is easily overlooked by clinicians.

Most patients diagnosed with late‐stage BMM may lose the opportunity to receive the normal dose and course of radiation and chemotherapy, which leads to a reduction in their survival.^3^, ^5^, ^6^, ^7^, ^8^, ^9^, ^10^ In addition, patients with symptomatic BMM have been excluded from previous clinical trials on BC. Thus, given the paucity of data in this area, the appropriate treatment of BC with BMM is not discussed in detail in the major guidelines.^11^, ^12^, ^13^ Though comprehensive therapies such as chemotherapy, endocrine therapy, molecular targeted therapy, and immunotherapy can improve the prognosis of patients with MBC,^14^, ^15^, ^16^, ^17^, ^18^ unfortunately, the prognosis of patients with BMM is still not ideal^3^, ^5^, ^6^, ^7^, ^8^, ^9^, ^10^ due to the lack of definitive treatments. Therefore, it is necessary to study BC with BMM to expand the understanding of this disease, provide effective diagnosis and treatment strategies, and further improve the survival of patients.

This study aimed at providing a reference for the diagnosis and treatment of MBC with BMM by investigating the clinical features, treatments, prognosis, and factors associated with MBC with BMM and by evaluating the factors that affect overall survival following BMM (BMMOS) and therapies after BMM.

## 2 METHODS

### 2.1 Patients

This is a retrospective study of the medical records of 3289 patients diagnosed with MBC and patients who developed metastatic disease during their follow‐up between June 1, 2010, and May 31, 2020, at the Affiliated Cancer Hospital of Zhengzhou University & Henan Cancer Hospital, Department of Breast Disease.

The inclusion criteria are as follows: (1) patients with invasive BC diagnosed pathologically; (2) non‐resectable local recurrence or MBC diagnosed at the Affiliated Cancer Hospital of Zhengzhou University & Henan Cancer Hospital between June 1, 2010, and May 31, 2020; (3) male BC or bilateral BC; and (4) known metastatic site. BMM was confirmed at our hospital through bone marrow aspiration smear and/or trephine biopsy. Patients were excluded if they had a history of other malignant tumors in the past 3 years, excluding cured cervical carcinoma in situ, skin basal cell carcinoma, or squamous cell carcinoma, or if there was incomplete information.

Patient information was recorded retrospectively through a database we established and updated, which includes demographics, clinical, and pathological characteristics, and treatment approaches. The patient's survival state was confirmed through outpatient, inpatient, or telephone‐based follow‐ups. The typical features of BMM recorded included irregular fever without chills during the course of the disease, fluctuation in body temperature between 37.5°C and 39.9°C, poor or no response to normal anti‐inflammatory treatment, and single lineage cytopenia, bi‐lineage cytopenia, or multilineage cytopenia with or without fever. Suspected BMM was confirmed using bone marrow aspiration smear and/or trephine biopsy. Patients with negative bone marrow smear results but high clinical suspicion of BMM underwent a repeat bone marrow trephine biopsy to confirm the findings. During the treatment, supportive care, such as transfusion of red blood cells and platelets and the administration of granulocyte growth factors, was provided. Further, the effect of treatment and hemogram parameters/body temperature change were regularly monitored. The latest follow‐up data were obtained on July 31, 2021.

### 2.2 Methods of diagnosis

**FIGURE 1:**
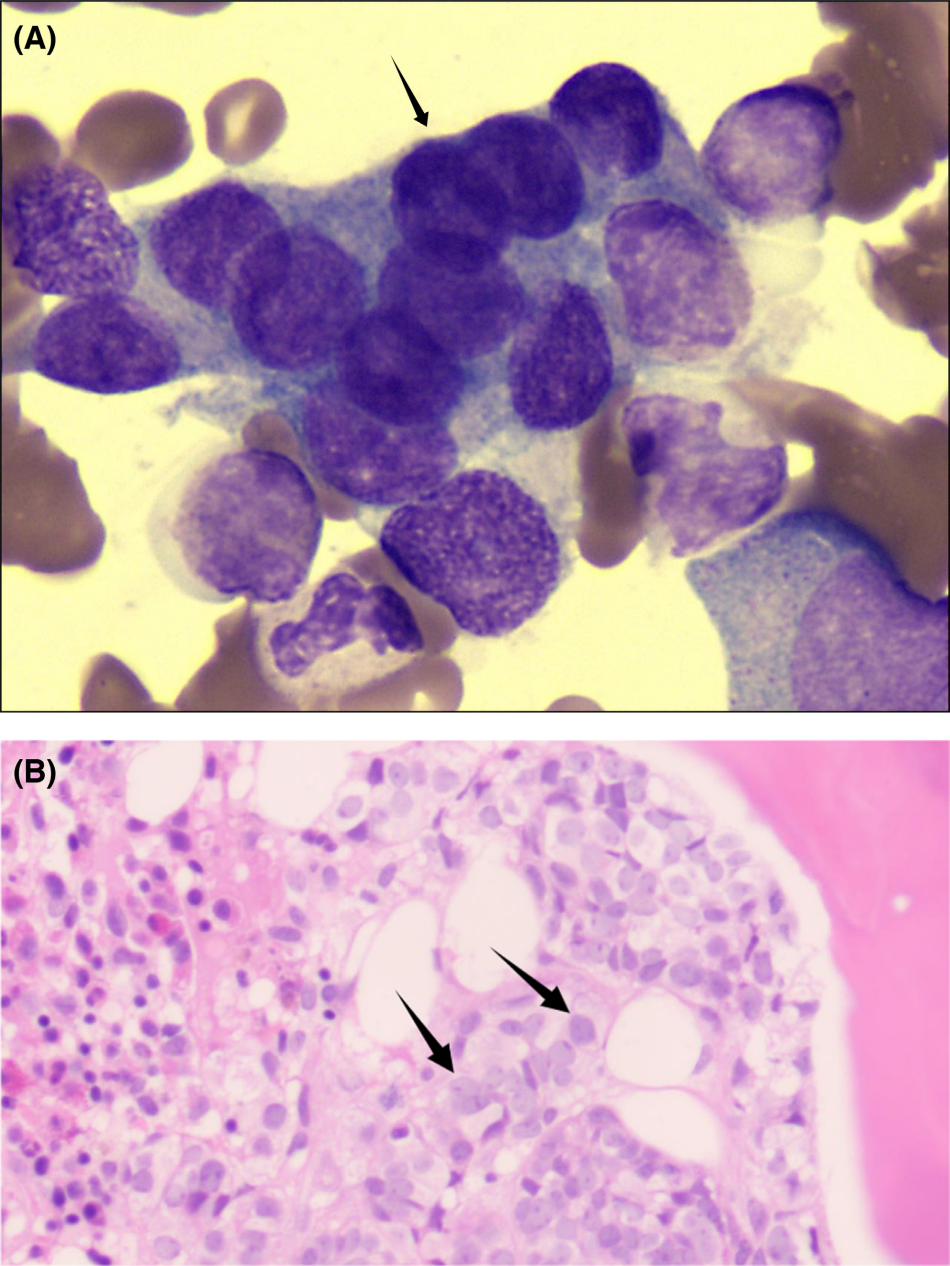
An example of the bone marrow aspiration smear and trephine biopsy results. (A) The image of bone marrow smear of a patient with breast cancer (Wright‐Giemsa staining, 10 × 100); (B) The photomicrograph of  bone marrow trephine biopsy of a patient with breast cancer (hematoxylin and eosin staining, 10 × 40).

All patients with BMM were diagnosed using bone marrow aspiration smear and/or trephine biopsy. Bone marrow aspiration smear and/or trephine biopsy was performed if the patient had irregular fever without chills during the disease course, no response to regular anti‐inflammatory treatment, continuous unexplained reduction in hemogram (hemoglobin, white blood cells [WBCs], platelets, etc.) that cannot be explained by blood loss, hemolysis, etc., myelosuppression more than grade 2 during chemotherapy, or prolonged recovery in the follow‐up period. In addition to bone marrow aspiration smear and/or trephine biopsy, peripheral blood smear tests were performed for patients who had single‐lineage cytopenia, bi‐lineage cytopenia, or multilineage cytopenia. Bone marrow aspirates or trephine biopsy specimens were extracted through the posterior or anterior iliac crest under local anesthesia, and approximately 0.1–0.2 mL of bone marrow aspirate was taken using a bone marrow aspiration package. Five to six smears were made in each case, and care was taken to ensure that bone marrow smears were labeled correctly and clearly. Each air‐dried smear was stained with Wright & Giemsa stain for 15 min. Trephine biopsy samples were preserved in 10% formalin solution and immediately transported to the central laboratory. BMM was diagnosed if one or more tumor cells were detected. A hematopathologist reconfirmed the BMM infiltration status by performing a bone marrow vessel count. The bone marrow aspiration smear and trephine biopsy of a confirmed case showed cells that were large in size, irregular in shape, rich in cytoplasm, and occurring in clusters. There were also visible vacuoles, irregular karyotypes, slightly rough, loose nuclear chromatin, and unclear nucleoli (Figure 1A,B).

### 2.3 Definition of terms

BMMOS was defined as the duration of overall survival from BMM diagnosis until death or the last follow‐up. BMM‐free interval was defined as the time from initial BC diagnosis to BMM diagnosis.

Treatment efficacy was classified according to the Response Evaluation Criteria in Solid Tumors version 1.1. The efficacy categories were complete response, partial response (PR), stable disease, and progressive disease (PD).

The degrees of fever and cytopenia were classified according to the National Cancer Institute Common Terminology Criteria for Adverse Events version 5.0.

### 2.4 Statistical analysis

SPSS (version 23.0; IBM Corp.) was used for statistical analysis. Differences between two variables were analyzed using the chi‐square test or Fisher's exact test. Nonparametric tests, such as Mann–Whitney *U* and Kruskal–Wallis, were used to compare the effect of a parameter between two multi‐sorted variables. The Kaplan–Meier method was used for survival analysis, and the log‐rank test was used for univariate analysis of prognosis among groups. Factors with a *p*‐value <0.15 in the univariate logistic regression were incorporated into the multivariate Cox proportional hazards regression model. The stepwise regression method was used in the analysis of the multivariate Cox proportional hazards model. Statistical significance was set at *p* < 0.05.

## 3 RESULTS

### 3.1 Clinical characteristics of BMM

**FIGURE 2:**
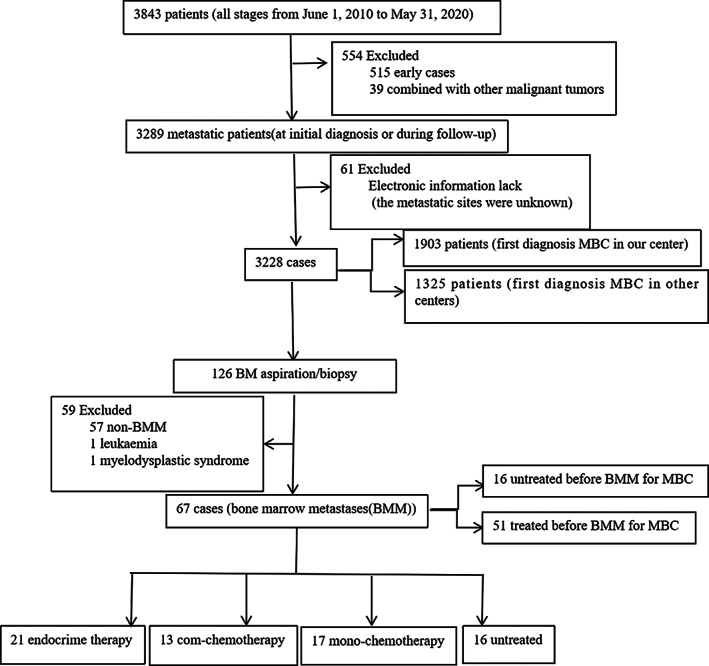
Schematic diagram of patient selection (n = 67).

A total of 3228 patients with MBC were admitted to the Affiliated Cancer Hospital of Zhengzhou University & Henan Cancer Hospital between June 1, 2010, and May 31, 2020. BMM was diagnosed in 67 (2.1%) patients, and they were included in this study (Figure 2). The median age was 48 years (range, 22–75), and the median disease‐free survival was 22.5 months (range, 0–180). Most patients with BMM had hormone receptor‐positive/human epidermal growth factor receptor 2‐negative (HR+/HER2−) tumors. Among the patients, 73.1%, 13.4%, and 13.4% had HR+/HER2− tumors, HER2+ tumors, and triple‐negative tumors, respectively. The most common symptoms of BMM were the coexistence of anemia and thrombocytopenia (26.9%), anemia (19.4%), and pancytopenia (17.9%). Anemia was mainly Grades 2–3, accounting for 80.6% of cases. Platelet counts were normal in 44.8% of the patients, and thrombocytopenia was mostly Grades 2–3, accounting for 41.8% of cases. Leukopenia was mostly Grades 0–1, accounting for 80.6% of cases. Fever was mostly Grades 0–1, accounting for 94.0% of cases. The patient body temperature fluctuated between 37.5°C and 39.9°C, without chills. The characteristics of the 67 patients are presented in Tables 1 and 2.

**TABLE 1:** Clinical characteristics of 67 patients with BMM and univariate analysis of BMMOS.

Characteristics	Patient (*n*)	Percentage	Median BMMOS (months)	χ^2^	*p*
Age (years)				2.460	0.117
<50	40	60.0	15.7		
≥50	27	40.0	5.2		
Sex				—	—
Female	66	98.5	15.7		
Male	1	1.5	5.2		
Menstrual status				0.422	0.516
Premenopause/Perimenopause	40	60.0	13.7		
Postmenopause	26	38.8	5.7		
Stage of initial diagnosis				9.732	0.021
I	2	3.0	1.7		
II	19	28.4	6.5		
III	21	31.3	7.2		
IV	25	37.3	9.7		
DFS (months)				0.017	0.897
<24	33	49.3	7.1		
≥24	34	50.7	9.7		
BMM‐free interval (months)				0.75	0.386
<36	35	52.2	7.6		
≥36	32	47.8	6.5		
Time between BM and BMM (months)				0.973	0.324
≤10	34	50.7	15.7		
>10	33	49.3	6.5		
Molecular type				5.722	0.057
HR+/HER2−	49	73.1	9.7		
HER2+	9	13.4	7.1		
TNBC	9	13.4	2.3		
Combined metastatic site				2.143	0.143
Viscera	44	65.7	18.6		
Non‐viscera	23	34.3	6.1		
Treatment for MBC before diagnosis of BMM				2.565	0.109
No	16	23.9	22.7		
Yes	51	76.1	6.5		
Endocrine therapy	36	53.7			
Chemotherapy	35	52.2			
Therapy at the diagnosis of BMM in cases with prior chemotherapy	35	52.2			
Chemotherapy	23	65.7			
Endocrine therapy	10	28.6			
Untreated	2	5.7			
Previous lines of therapies for metastatic disease				2.779	0.249
0	16	23.9	22.7		
1–2	32	47.8	7.2		
≥3	19	28.3	5.7		
With or without fever				0.402	0.526
No	55	82.1	6.1		
Yes	12	17.9	9.7		
WBC (×10^9^/L) count				0.372	0.541
Normal	43	64.2	8.6		
Low	24	35.8	7.2		
Hemoglobin (g/L)				1.639	0.200
≥80	38	56.7	8.6		
<80	29	43.3	5.7		
PLT (×10^9^/L) count				8.613	0.003
≥75	34	50.7	16.6		
<75	33	49.3	4.9		
Therapy after BMM				20.102	<0.001
Endocrine therapy	21	31.3	15.7		
Com‐chemotherapy	13	19.4	9.7		
Mono‐chemotherapy	17	25.3	8.6		
Untreated	16	23.9	2.9		

Abbreviations: BM, bone metastases; BMM, bone marrow metastases; BMMOS, overall survival following diagnosis of bone marrow metastases; DFS, disease‐free survival; HER2, human epithelial growth receptor‐2; HR, estrogen receptor; MBC, metastatic breast cancer; PLT, platelet; TNBC, triple‐negative breast cancer; WBC, white blood cell.

**TABLE 2:** Symptom classification in the diagnosis of BMM.

Characteristics	Patient (*n*)	Percentage
Fever		
Grade 0	55	82.1
Grade 1	8	11.9
Grade 2	4	6.0
Anemia		
Grade 0	11	16.4
Grade 1	2	3.0
Grade 2	25	37.3
Grade 3	29	43.3
Leukopenia		
Grade 0	43	64.2
Grade 1	11	16.4
Grade 2	11	16.4
Grade 3	2	3.0
Thrombocytopenia		
Grade 0	30	44.8
Grade 1	4	6.0
Grade 2	13	19.4
Grade 3	15	22.4
Grade 4	5	7.4
Symptom of initial diagnosis BMM		
Fever only	3	4.4
Anemia only	13	19.4
Thrombocytopenia only	1	1.5
Leukopenia only	2	3.0
Anemia + thrombocytopenia	18	26.9
Anemia + leukopenia	4	6.0
Thrombocytopenia + leukopenia	3	4.4
Pancytopenia	12	17.9
Pancytopenia + fever	1	1.5
Fever + anemia	4	6.0
Fever + anemia + thrombocytopenia	2	3.0
Fever + anemia + leukopenia	2	3.0

Abbreviation: BMM, bone marrow metastases.

At MBC diagnosis, the bones were the first metastatic sites in 52 (77.6%) cases. The time from BM to BMM ranged from 0 to 104.0 months, and the median time was 17.0 months. Nine patients (13.4%) had BMM and BM simultaneously, whereas the other 58 (86.6%) patients had BMM after BM. The BMM‐free interval ranged from 0 to 218.4 months, and the median BMM‐free interval was 52.1 months.

### 3.2 Patient treatment approaches after BMM diagnosis

The median prior therapy line was two lines for MBC (range, 0–5). Regarding treatment after BMM, 21 patients received endocrine therapy. Among them, three patients were treated with a combination of endocrine therapy and cyclin‐dependent kinase 4/6 (CDK4/6) inhibitors. Eight patients were treated with aromatase inhibitors, and five patients were treated with fulvestrant. The rest of the patients were treated with medroxyprogesterone. Thirteen patients received combined chemotherapy, among whom seven received taxane‐based chemotherapy, while six received vinorelbine‐based chemotherapy. Seventeen patients were treated with mono‐chemotherapy, 13 of whom received taxane monotherapy (two patients with HER2+ tumors were treated with taxane + trastuzumab + pertuzumab regimen), while four were treated with single capecitabine monotherapy. Sixteen patients remained untreated.

### 3.3 BMMOS and factors affecting BMMOS

**FIGURE 3:**
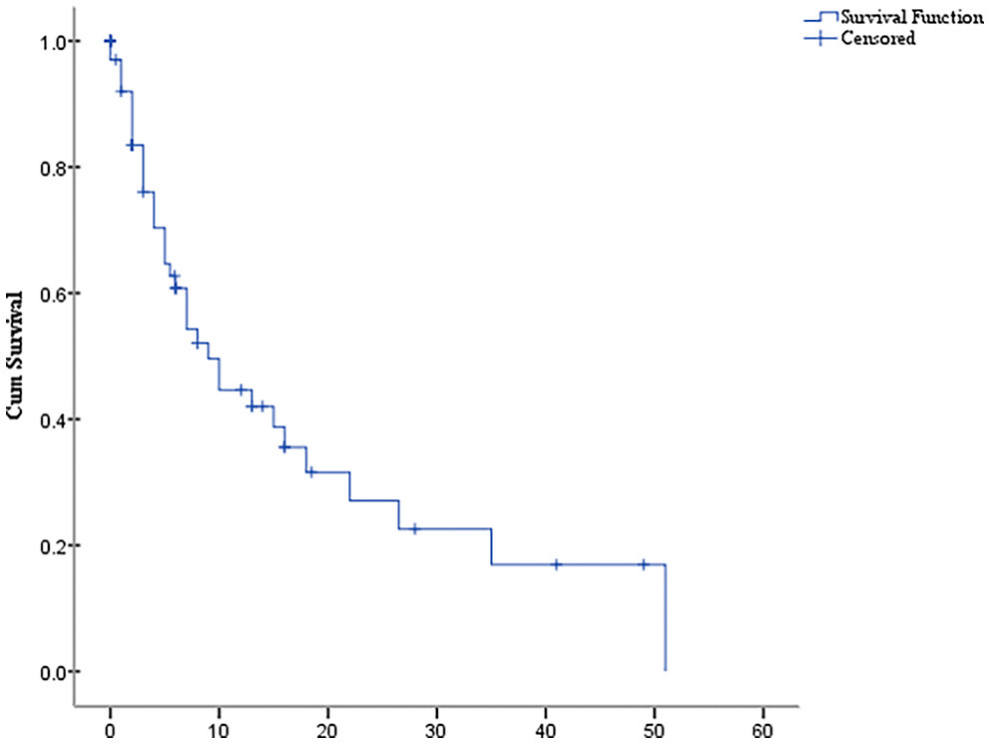
The Kaplan–Meier curve. Overall survival time following bone marrow metastasis.

At the time of the latest follow‐up, 51 (76.1%) patients had died (the detailed time of death was unknown for 6 patients), 12 (17.9%) patients survived, and 4 (6.0%) patients were lost to follow‐up. The median BMMOS was 7.6 months (95% CI, 3.9–11.3) (Figure 3). There was no significant difference in the BMM‐free interval between <36 months and ≥36 months (χ^2^ = 0.75, *p* = 0.386). There was no significant difference in the molecular types HR+/HER2−, triple‐negative, and HER2+ (χ^2^ = 5.722, *p* = 0.057). There was no significant difference between the presence or absence of fever (χ^2^ = 0.402, *p* = 0.526), between normal or abnormal WBC count (χ^2^ = 0.372, *p* = 0.541), and between hemoglobin levels ≥80 or <80 g/L (χ^2^ = 1.639, *p* = 0.200). In contrast, there was a significant difference between platelet counts ≥75 × 10^9^/L and <75 × 10^9^/L (χ^2^ = 8.613, *p* = 0.003) (Table 3).

**TABLE 3:** Baseline grade of hemogram and fever among different treatments at the diagnosis of BMM.

	PLT Grade1	PLT Grade2	PLT Grade3	PLT Grade4	HB Grade1	HB Grade2	HB Grade3	WBC Grade1	WBC Grade2	WBC Grade3	Fever Grade1	Fever Grade2
ET	2	4	2	2	0	9	8	4	3	1	1	1
Mono‐CT	0	2	4	0	0	4	7	3	0	1	4	2
Com‐CT	2	4	3	2	0	9	6	2	4	0	2	1
Untreated	0	3	6	1	2	3	8	2	4	0	1	0

Abbreviations: BMM, bone marrow metastases; Com‐chemotherapy, combined therapy; ET, endocrine therapy; HB, hemoglobin; Mono‐CT, mono‐chemotherapy; PLT, platelet; WBC, white blood cell.

**FIGURE 4:**
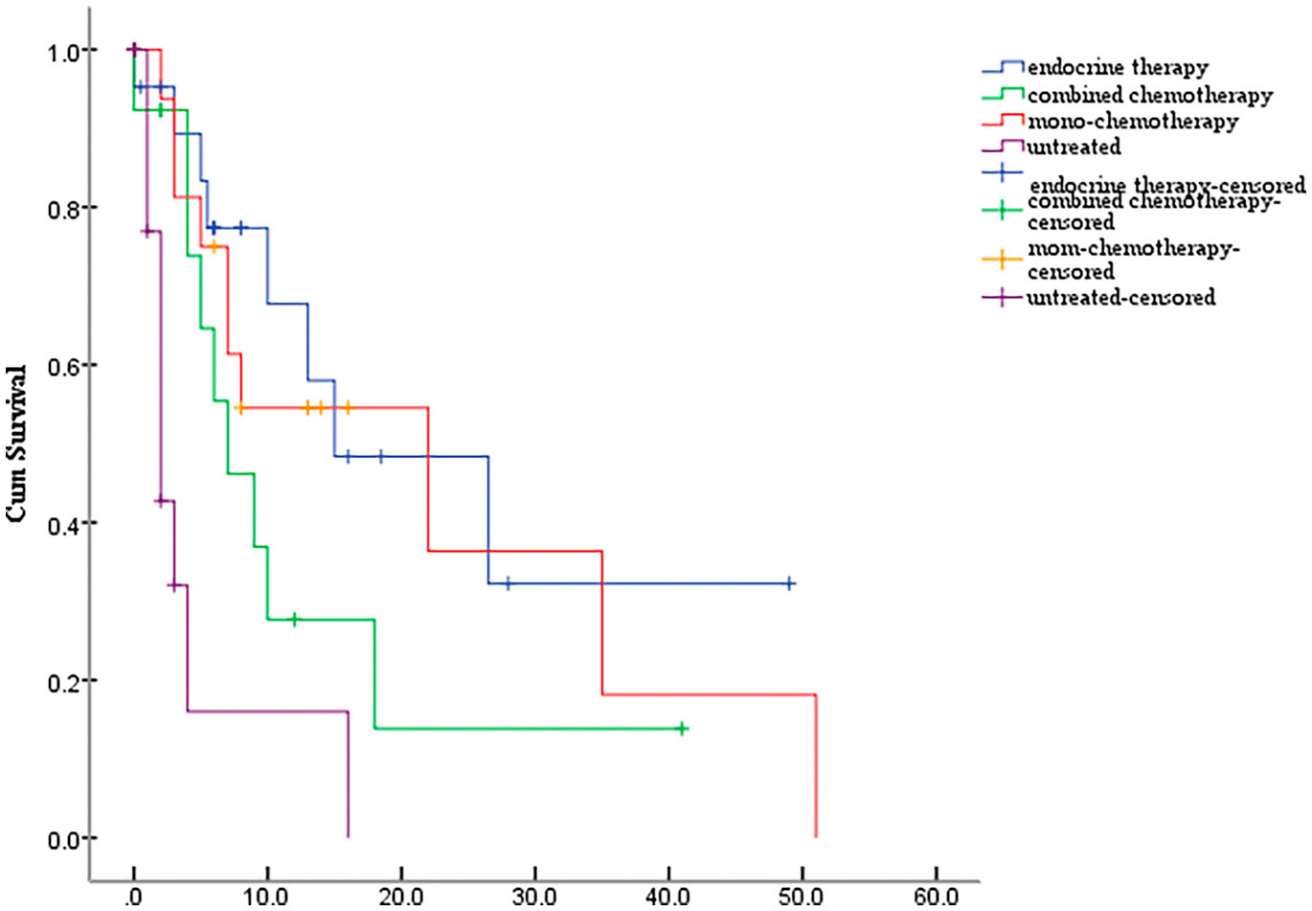
The Kaplan–Meier curve. Effects of different treatments on overall survival time following bone marrow metastasis.

The BMMOS of patients who received endocrine therapy, combined chemotherapy, and mono‐chemotherapy after BMM was 15.7, 9.7, and 8.6 months, respectively, whereas that of untreated patients was 2.9 months. The difference among the results was statistically significant (χ^2^ = 20.102, *p* < 0.0001). The effects of the different treatments on BMMOS are shown in Figure 4 and Table 3.

Factors with a *p*‐value <0.15 in the univariate analysis were entered into a multivariate Cox regression model. Such factors include age, stage, molecular subtype, metastatic sites (visceral or nonvisceral), with or without therapy before BMM, platelet count, and treatment after BMM. The results showed that age, platelet count, and treatment after BMM affected BMMOS (Tables 1 and 4).

**TABLE 4:** Multivariate analysis of factors associated with BMMOS.

	B	*p*	CI (95%)
Age	−0.875	0.008	0.417 (0.218–0.798)
Thrombocytopenia	−1.059	0.002	0.347 (0.180–0.668)
Therapy after BMM		<0.001	
Therapy after BMM‐ET	−1.678	<0.001	0.187 (0.078–0.445)
Therapy after BMM‐Com‐CT	−1.457	0.002	0.233 (0.093–0.585)
Therapy after BMM‐Mono‐CT	−1.969	<0.001	0.140 (0.057–0.345)

Abbreviations: BMM, bone marrow metastases; BMMOS, overall survival following diagnosis of bone marrow metastases; Com‐chemotherapy, combined therapy; ET, endocrine therapy; Mono‐CT, mono‐chemotherapy.

### 3.4 Hemogram parameters and/or body temperature change of 68 regimens after BMM

The 67 patients with BC and BMM underwent a total of 119 therapeutic regimens after BMM. Sixty‐eight regimens with efficacy evaluation confirmed by imaging examinations, and simultaneous evaluation of body temperature and/or hemogram parameters, were included. Of these regimens, 38 regimens improved the hemogram parameters and/or body temperature, 10 regimens worsened body temperature and/or hemogram parameters, while the remaining 20 regimens did not affect body temperature and/or hemogram parameters (Table 5).

**TABLE 5:** The relationship between change of hemogram and/or body temperature and the efficacy confirmed by imaging examinations after BMM.

Efficacy confirmed by imaging examinations	Change of hemogram and/or body temperature		
	Improved	Unchanged	Deteriorative
PR	13	1	0
SD	15	10	2
SD ≥6 months	9	0	0
PD	1	9	8

Abbreviations: BMM, bone marrow metastases; PD, progressive disease; PR, partial response; SD, stable disease.

Among the 14 patients with PR confirmed by imaging examinations, 13 cases showed improvements in hemogram parameters and/or body temperature. Likewise, in the 18 cases which showed PD confirmed by imaging examinations, the hemogram parameters and/or body temperatures deteriorated in eight cases and remained unchanged in nine cases (Table 5). Changes in patient hemogram parameters and/or body temperature during treatment were consistent with the efficacy confirmed by imaging examinations (*p* < 0.0001).

## 4 DISCUSSION

To the best of our knowledge, the current study is the one with the largest sample size to describe the clinicopathological features, prognosis, and therapy of BC with BMM. In our study, all the patients had BM complications. In 86.6% of the cases, BMM was diagnosed after BM, while in the remaining 13.4%, BMM was diagnosed simultaneously with BM. The findings of the current study are consistent with those of previous reports.^8^, ^9^ BMM often has no obvious symptoms, unless it causes severe fever, anemia, or bleeding.^3^, ^4^, ^5^, ^6^, ^8^, ^9^, ^10^ BC with BMM is not common in clinical practice, and many clinical doctors do not have a clear or comprehensive understanding of the disease. In addition, bone marrow trephine biopsy is not a routine examination for BC and has a certain proportion of dry taps. These may be the reasons why BMM is generally difficult for doctors to detect, thus leading to delayed diagnosis and underestimation of the disease.^19^ Furthermore, because BM causes bone pain or elevation in alkaline phosphatase (ALP) levels^20^ and because BMM was diagnosed simultaneously with or after BM, the relationship between bone pain or ALP level elevation in patients and BC with BMM needs further exploration, which was different from the investigation in previous studies.^6^, ^7^, ^9^

The most common clinical manifestations observed in the present study were the coexistence of anemia and thrombocytopenia, followed by anemia only, and pancytopenia. Cases of Grade 3 or higher leukopenia were rare at 3.0% (2/67), and a diagnosis of BMM with leukopenia only was also rare at 3.0% (2/67). Leukopenia was mostly grades 0–1, accounting for 80.6% of cases. Fever without chills was mostly Grades 0–1, accounting for 94.0% of cases. These findings were consistent with those of previous reports.^3^, ^4^, ^5^, ^6^, ^8^, ^9^, ^10^ The results of the current study showed that patients with BMM generally had low‐grade fever without chills and normal WBC counts, and those with leukopenia only had a small probability of BMM. Similarly, low‐grade fever was also reported in three previous studies.^4^, ^6^, ^9^ Considering these results, if there is an unexplained reduction in hemogram parameters, especially anemia and/or thrombocytopenia, and/or unexplained fever without chills among BC patients with BM, BMM should be suspected. In addition, five patients in this study received a BMM diagnosis after two bone marrow aspiration smears/biopsies, which suggests that multiple bone marrow aspiration smears/biopsies should be recommended for patients with suspected BMM.

The results of the univariate and multivariate analyses showed that BMMOS was associated with platelet count. In contrast, BMMOS had less correlation with a reduction in the WBC count and hemoglobin level, which was only reported in one previous study.^5^ It is well known that thrombocytopenia recovers slowly, and treatment is stymied by the immaturity of current drugs to treat thrombocytopenia and the absence of specific recommendations to prevent thrombocytopenia.^21^ A possible reason for this may be that BMM affects the hematopoietic function of the bone marrow and results in decreased tolerance to chemotherapy, which ultimately leads to a short survival time.^5^ Trilaciclib, an intravenous CDK4/6 inhibitor, demonstrated an improvement in the patient's chemotherapy tolerability, as shown by myelopreservation across multiple hematopoietic lineages resulting in fewer supportive care interventions and dose reductions. Whether or not trilaciclib plays a role in BC with BMM needs further investigation.^22^

In the current study, we found that 73.1% of the patients with BMM had HR+/HER2− tumors, which was consistent with the results of previous studies.^8^, ^9^ Various guidelines^11^, ^12^, ^13^ recommend endocrine therapy as the preferred treatment for patients with advanced HR+ BC unless there are concerns regarding a life‐threatening disease or endocrine resistance. We also found that, similar to patients without BMM, patients with HR+/HER2− BMM could benefit from endocrine therapy (BMMOS, 15.7 months). Currently, endocrine therapy and targeted therapy^17^, ^23^, ^24^, ^25^, ^26^, ^27^, ^28^, ^29^, ^30^, ^31^, ^32^ aimed at HR+/HER2− tumors further improve the prognosis of patients. However, no study has confirmed the benefit of this therapeutic strategy in patients with visceral metastases. The RIGHT Choice study (NCT03839823) is exploring the combination of endocrine therapy and a CDK4/6 inhibitor in comparison with chemotherapy in the setting of significant visceral impairment, and the results will answer this question. At present, expert consensus suggests^33^ that patients with extensive BMM and poor clinical chemotherapy tolerance could be treated with CDK4/6 inhibitors combined with endocrine drugs.

We found that the BMMOS of patients who underwent treatment after the diagnosis of BMM was significantly longer than that of untreated patients (2.9 months, *p* < 0.0001).^3^, ^6^, ^8^, ^9^, ^10^ Patients with BMM have significant hemogram abnormalities, and chemotherapy that leads to bone marrow suppression may further aggravate bone marrow abnormalities. These increase the risk associated with chemotherapy, which may be the reason that clinicians are reluctant to administer aggressive chemotherapy. However, bone marrow suppression is caused by tumor invasion, and treatment can only briefly improve the levels of the blood cells by destroying the tumor cells. In this study, the BMMOS of patients who underwent mono‐chemotherapy and combined chemotherapy were 8.6 and 9.7 months, respectively. Thus, after the diagnosis of BMM in chemo‐sensitive patients, rather than rejecting chemotherapy because of an abnormal hemogram, a highly efficient chemotherapy regimen that has mild side effects and good tolerability, for example, utidelone,^34^ programmed cell death protein 1 (PD‐1),^16^ programmed death‐ligand 1 (PD‐L1),^35^ or bevacizumab,^36^ should be chosen.

This study also revealed that the improvement or deterioration of body temperature and hemogram parameters during treatment was consistent with the overall effect confirmed by imaging examinations (*p* < 0.0001). In other words, when the treatment is effective, the patient's body temperature and/or hemogram parameters improves or returns to normal, and as the disease progresses, fever and/or cytopenia will reappear. Thus, hemogram parameters and/or body temperature change may be indicators of treatment efficacy, especially in BC patients combined with BM only. Knowing this, clinicians can then adjust the treatment plan to prevent delayed treatment.

This study had some limitations. First, the baseline characteristics of patients treated with different therapies were not consistent. Thus, it was not possible to accurately compare the treatment efficacy, and further verification is necessary. Next, the total number of patients in the study was small, and the number of patients assigned to each molecular subtype was relatively low. Thus, there was a low statistical power for some data, and further investigation is therefore required to obtain more data and higher statistical power. In addition, the diagnosis of BMM is dependent on the performance of a bone aspiration smear and/or trephine biopsy, which was not routinely done. Therefore, the study included intrinsic selection and indication bias. Moreover, we were unable to explore the efficacy of other treatment methods, such as CDK4/6 inhibitors, novel chemotherapeutic drugs, antibody‐drug conjugate drugs, anti‐HER2 therapy, and PD‐1/PD‐L1, on BMM and this needs further exploration. Additionally, further exploration of the underlying mechanism of BMM, including the roles of bone marrow mammaglobin‐1 (SCGB2A2)^37^ or miRNA‐1231 in exosomes^38^ in BC with BMM, is needed. This study is also limited by the fact that it spans a 10‐year period and the updated treatment methods will affect the choice of treatment strategies and BMMOS.

## 5 CONCLUSIONS

BMM should be considered in BC patients with BM if an unexplained reduction in hemogram parameters, especially anemia and thrombocytopenia, and/or fever without chills occur. Active, effective, and individualized treatment strategies can prolong BMMOS. When formulating treatment plans for patients, consistent with the metastasis treatment strategies for other sites, we need to comprehensively select active and individualized treatment plans for patients according to their molecular type, previous treatment plans, sensitivity to drug treatment, and adverse drug treatment reactions. Our findings may assist clinicians with assessing the characteristics, diagnosis, treatment, and prognosis of MBC with BMM.

## AUTHOR CONTRIBUTIONS

**Limin Niu:** Conceptualization (equal); data curation (equal); methodology (equal); resources (equal); software (equal); visualization (equal); writing – original draft (equal); writing – review and editing (equal). **Huimin Lv:** Data curation (equal); formal analysis (equal); methodology (equal); resources (equal); software (equal); supervision (equal); validation (equal); writing – review and editing (equal). **Mengwei Zhang:** Formal analysis (equal); methodology (equal); resources (equal); validation (equal); writing – review and editing (equal). **Huiai Zeng:** Data curation (equal); formal analysis (equal); resources (equal); software (equal); writing – review and editing (equal). **Shuzhen Fu:** Data curation (equal); resources (equal); writing – review and editing (equal). **ShuDe Cui:** Conceptualization (equal); project administration (supporting); visualization (equal); writing – review and editing (supporting). **Zhenzhen Liu:** Conceptualization (equal); project administration (supporting); visualization (equal); writing – review and editing (supporting). **Min Yan:** Conceptualization (equal); methodology (equal); project administration (equal); resources (equal); supervision (equal); writing – review and editing (lead).

## FUNDING INFORMATION

No specific funding was received.

## CONFLICT OF INTEREST STATEMENT

The authors declare that they have no conflict of interest.

## ETHICAL APPROVAL

The authors are accountable for all aspects of the work and ensuring that questions related to the accuracy or integrity of any part of the work are appropriately investigated and resolved. This study was approved by the medical ethics committee of Henan Cancer Hospital (no. 2017407). Informed consent from patients was waived owing to the retrospective nature of this study. All procedures performed in this study, involving human participants, are in accordance with the Declaration of Helsinki (as revised in 2013).

## Data Availability

Availability of data and material Available.
